# It’s not just FaceTime: core competencies for the Medical Virtualist

**DOI:** 10.1186/s12245-019-0226-y

**Published:** 2019-03-12

**Authors:** Rahul Sharma, Sapir Nachum, Karina W. Davidson, Michael Nochomovitz

**Affiliations:** 1 NewYork-Presbyterian-Weill Cornell Medicine, New York, NY USA; 2 Weill Cornell Medicine, New York, NY USA; 3 Northwell Health, New York, NY USA

**Keywords:** Telemedicine, Education, Certification, Competencies

## Abstract

New applications for virtual healthcare have resulted in an expansion of medical care beyond traditional healthcare settings. However, the rapid development of telemedicine as a field has resulted in poor standardization of the care provided through this new medium. The authors outline core competencies to be used in developing training programs for practicing physicians, medical students, and other clinicians using telemedicine as a medium for providing patient care. These competencies aim to provide a framework for defining a standard of care in telemedicine and the ultimate development of a certification in the field.

## Background

The exponential growth in the use of digital devices and digital-savvy patients’ demand for convenience and cost savings is driving the adoption of and demand for virtual health ahead of the maturation of training and standards. Core competencies and curricula for medical virtualism have evolved from early adopters, but have been based on experiential learning. It is incumbent on those at the edge of new technologies and services to share their experiences to advance a new field. New use cases, guidelines, and expectations are being learned in real time with more experience across specialties. Physicians and other healthcare providers with the most experience are sharing their knowledge at national meetings, mini-courses, and in publications, both peer-reviewed and in the lay press. There is a similarity with the evolution of other new disciplines like geriatrics and critical care that now have their own curricula that follow training in internal medicine. Now is the time to launch medical virtualism accreditation. We recommend a formal investigation into the core competencies needed, the innovative education training and delivery systems to be used, and the learners who need this formal recognition of their competency. We propose the competencies in this document as the basis for national discussion and look to hold a summit on the topic based on academic medicine and industry partners.

Virtual health, telehealth, or telemedicine has only recently entered the mainstream of medical practice, care delivery, and medical literature, with an explosion of recently presented concepts, use cases, and models of care [1, 2]. This evolving practice broadly includes digital communication by clinicians, supported by homecare, emergency services, and remote monitoring. The field has shifted from focusing on minor medical illness to harnessing the technology for management of chronic and more complex conditions, particularly with regard to global payment models. Virtual health is now incorporated into the key strategic plans for most major medical centers, physician organizations, payers, and healthcare providers, with initiatives often originating in or led by emergency departments [3].

The assumption that everyday lifestyle technologies, such as FaceTime®, can prepare physicians and healthcare providers for virtual care is fallacious. The diversity of platforms and providers has led to documented inconsistencies in the care provided via virtual health services [4]. The maturation of virtual health as a discipline requires the standardization of core competencies for clinicians, as this new paradigm goes far beyond replacing a traditional clinician encounter and ushers in a new generation of medical practice. We have developed a curriculum including both didactic and simulation components based on our own experience and subsequently introduced a telemedicine elective for medical students. We will now include Virtual Healthcare as part of the required curriculum for all incoming medical students. We anticipate that these competencies will serve as a framework for certification in the field of virtual care as an evolving discipline integrated into the care continuum.

## Main text

Licensed medical professionals are trained for the conventional practice of medicine. However, virtual healthcare adds new layers of complexity requiring competencies beyond those currently expected of all physicians [5]. A number of organizations in the public and private sector appear to be structuring their own content focused on the specific needs of their clinical programs [6]. We propose a standardization of the training for telemedicine providers and have developed a set of core competencies divided into *three domains* (Table 1). We expect that clinicians planning on practicing substantially as medical virtualists will need to demonstrate a set of competencies through written and simulated scenarios.

**Table 1:** Core competencies for virtual healthcare

Competencies		Differences between bedside practice and virtual care	Helpful resources for curriculum development
Domain I: Digital communication and webside manner	Optimal visualization, body language, and speech	*Communication speed*—reduced for clear enunciation to ensure clarity over online platforms. *Colloquial speech*—avoided to differentiate between professional encounter and lifestyle video communication such as FaceTime® *Body motion and gestures*—minimized and made in full view of camera. *Motions* should be slowed to avoid blurring or poor visualization over video. *Background, lighting, and framing* are essential components of a virtual encounter which differ from traditional encounters. *Dress*—solid clothes with a neutral background project optimally in a virtual setting. *Camera*—located in a fixed position with clinician’s head and shoulders centered. Clinicians look at the camera rather than screen to maintain “eye contact.”	Media training groups such as Media Training Worldwide: https://www.tjwalker.com Conferences offer simulation based training for clinicians: https://www.virtualhealthcarenyc.com The American Telemedicine Association offers courses and webinars: http://learn.americantelemed.org/diweb/start Coordinator training modules: http://www.caltrc.org/knowledge-center/training/
	Graphic-assisted communication	*Imaging and diagnostic findings*—conveyed to patients using a screen share methodology	
	Virtual technologies	*Understand*—be familiar with virtual health platforms *Troubleshoot*—from both patient and clinician perspective	Systems vary by vendor. Require in-servicing and helpline access. Varies by provider (hospital, medical group, insurance company, employer).
Domain II: Scope and standards of care	Licensing	*State-specific licensing requirements***—**limitations on patient location and physician licensing built into each program.	Telemedicine licensing requirements: http://www.fsmb.org/siteassets/advocacy/key-issues/telemedicine_policies_by_state.pdf Cross-state licensing: https://www.ama-assn.org/practice-management/digital/cross-state-licensing-process-now-live-8-states
	Billing and insurance	*Coverage for virtual visits*—varies by locale and insurance carrier. Medicare policy ongoing evolution. Requires regular updating.	CMS Telemedicine services: https://www.cms.gov/medicare/medicare-general-information/telehealth/telehealth-codes.html Reimbursement Laws: https://www.cchpca.org/telehealth-policy/current-state-laws-and-reimbursement-policies
	HIPAA compliance	*Privacy***—**communicate with patient about the privacy of their location. Physician telemedicine visits should be conducted from appropriate space with the necessary privacy.	
	Prescribing	*Legal limits of e-prescribing*—for both controlled and uncontrolled substances over virtual platforms.	
	Virtual care pathways	*Appropriate follow-up***—**provide summation, instruction for treatment and follow-up (including home care or ambulatory diagnostic services), and precautions. *Emergent response***—**virtual visits may require activation of emergency services. Knowledge of patient location and ability to deploy EMS.	Record patient address during intake. Maintain list of emergency and urgent care centers in area serviced by telemedicine program. Telemedicine applications course: https://telemedicine.arizona.edu/training.cfm
Domain III: Virtual clinical interactions	Environmental assessment	*Safety, cleanliness, activities of daily living*—environment provides additional information beyond traditional patient encounter	
	Virtual physical exam	*Remote exam techniques***—**physician-guided or caregiver-assisted patient examinations used to assist in diagnostic accuracy. Alternatively, additional on-site providers (EMS, nursing) may assist if present. *Remote monitoring devices*—use of home blood pressure cuffs, smart watches, and glucometers for data gathering	To date, little research exists on these techniques. Evidence remains largely anecdotal and experience based including telemedicine physical exam techniques (http://www.telemedmag.com/article/telemedicine-physical-better-think/) and physician-guided patient self-examination case report (https://www.liebertpub.com/doi/full/10.1089/tmj.2018.0115).
	Group interactions	*Management of group interactions*—focus on family and group dynamics. Ensure HIPPA compliance in advance. Establish goals in advance with care management team. Observe experienced virtual health practitioner	

This table outlines proposed core competencies for physicians providing care via telemedicine and includes resources for curricular development and continued education

## Conclusions and next steps

What is the optimal pathway to accomplish consensus on core competencies for medical virtualism? In other practice areas, we have followed various leisurely pathways. Typically, groups of practitioners and experts start to call for standards in an area. Consensus grows that there is need for additional, standardized training. Those who already have been practicing in the specific area are consulted, and consensus coalesces around the need for formally recognized competencies. Frequently, professional societies are formed, and they start to generate consensus papers on optimal training and training modules.

Nearly 2 years ago, the American Medical Association encouraged the accrediting bodies for both undergraduate and graduate medical education to include core competencies for telemedicine in their programs [7]. It is time to accelerate this timeline. How shall we ensure that medical virtualist core competencies follow the most efficient, transparent, and equitable path to training and dissemination for the many physicians and healthcare providers who will practice this way, today? Ideally, an independent, highly respected, national, non-governmental institution would convene a roundtable on medical virtualism, and do so expeditiously. The invitees should include experts from a variety of disciplines, such as biomedical informatics, ethics, medical education, as well as the medical specialties already using this approach and the accrediting organizations involved in training our future and current physician workforces. Topics to be considered are not only which core competencies to include, but how to test for competence, how to minimize harms and unintended consequences that could result from this new medical practice, and how to assess quality in this area. The white paper resulting from this type of open and national dialogue could then be posted for public comment, and demonstration training projects conducted. There likely will need to be a professional society to ensure ongoing dialogue and increasing sophistication in the type, quality, and domains of medical practice that can and should be delivered virtually.

Every passing year sees more use cases of virtual care described—the once sporadic use of the virtual medium by organizations and their clinicians is becoming ubiquitous [8]. This movement of the practice of medicine into a new sphere of virtual care will require a large cohort of clinicians to practice as medical virtualists on a fulltime basis or be acquainted with the medium and qualified to practice for periodic use. A structured training and certification program for current practitioners and all medical students is an imperative to ensure high-quality virtual care.
